# The Person’s Care Requires a Sex and Gender Approach

**DOI:** 10.3390/jcm10204770

**Published:** 2021-10-18

**Authors:** Ilaria Campesi, Andrea Montella, Giuseppe Seghieri, Flavia Franconi

**Affiliations:** 1 Laboratory of Sex-Gender Medicine, National Institute of Biostructures and Biosystems, 07100 Sassari, Italy; franconi.flavia@gmail.com; 2 Department of Biomedical Sciences, University of Sassari, 07100 Sassari, Italy; montella@uniss.it; 3 Department of Epidemiology, Regional Health Agency of Tuscany, 50124 Florence, Italy; gseghieri@tin.it

**Keywords:** sex–gender, social aspects, interdisciplinary, caregiver, doctors’ prescribing patterns

## Abstract

There is an urgent need to optimize pharmacology therapy with a consideration of high interindividual variability and economic costs. A sex–gender approach (which considers men, women, and people of diverse gender identities) and the assessment of differences in sex and gender promote global health, avoiding systematic errors that generate results with low validity. Care for people should consider the single individual and his or her past and present life experiences, as well as his or her relationship with care providers. Therefore, intersectoral and interdisciplinary studies are urgently required. It is desirable to create teams made up of men and women to meet the needs of both. Finally, it is also necessary to build an alliance among regulatory and ethic authorities, statistics, informatics, the healthcare system and providers, researchers, the pharmaceutical and diagnostic industries, decision makers, and patients to overcome the gender gap in medicine and to take real care of a person in an appropriate manner.

## 1. Introduction

### 1.1. Definition of Sex and Gender

Sex and gender are often used interchangeably, but they are not synonyms. In general, sex is confined to the biological body [1,2,3,4,5]. Genes and sexual hormones have pivotal roles in determining male and female phenotypes [6,7,8,9], leading to the modification of enzymes, transporters, receptors, and other pharmacological targets or of their activities through numerous mechanisms [10,11,12]. The definition of gender is more complex, as testified by the numerous gender definitions available (Table 1). Gender includes socioeconomic status, income, education, neighborhood characteristics, lifestyles, environmental exposures including drugs, access to healthcare, and other social determinants of health [13]. Life experiences such as smoking can modify male and female phenotypes [14,15]. Numerous aspects of gender are changeable over time being different among countries and cultures. It is very hard to separate sex and gender because they interact with each other in a continuous multidimensional entangled manner. Some authors have proposed to use the term sex–gender-based medicine [16,17]. Here, we used the term sex–gender-based because it recognizes the value of both the biological and social–cultural–economic context.

### 1.2. Sex–Gender-Based Medicine: Historical Backgrounds

In the era of evidence-based medicine, the patient is not considered individually, but as a member of a group, and mainly as a male member. However, this vision presents several pitfalls [19]. Today, the unique biological characteristics of the individual or a group of individuals are increasingly being considered [20,21] to reach a more precise diagnosis and therapy (personalized medicine). However, there are still some knowledge gaps because the recognition of a person requires the recognition of sex–gender aspects. 

Several disparities in diagnosis, therapy, and outcomes can be ascribed to the lack of investigations in female animals and women [22,23,24]. Lack of good-quality results creates a bias that particularly affects women, who have historically been neglected in clinical research except for studies on the reproductive system [25].

To elevate the recruitment of women and other minorities, the USA produced the Revitalization Act of 1993, which requires the enrolment of women and minorities in clinical trials funded by the NIH [26]. Later, NIH further strongly recommended the inclusion of sex as a biological variable [27,28,29]. Even later, the Canadian Institutes of Health Research mandated sex and gender-based analysis, which recognizes intersectionality. Its awareness leads to a better knowledge of the differences among us [30]. Finally, last year, Horizon Europe indicated the need for intersectional analyses in gender and sex studies [31]. Notably, the sex–gender approach is also necessary to adhere to the suggestions of WHO documents “Health for All”, “Ottawa Charter for Health Promotion”, and “People Centred Health Care” [32,33,34]. 

Despite these mandates and recommendations, a recent survey in neuroscience observed little progress in sex and gender research. In particular, there was a 30% elevation of manuscripts that included both men and women, but only 19% had an appropriate design for sex and gender studies, and only 5% analyzed sex as a discovery variable versus 2% in 2009 [31]. In addition, from 1966 to 2018, in 7 (especially HIV/AIDS, chronic kidney diseases, and cardiovascular diseases) of 11 disease categories, there was sex bias in the enrollment and that has not changed over recent years but improved compared to before or during 1993 [22].

Globally, the above data indicate that sex and gender bias against female participants in clinical studies persists, despite legal and policy initiatives to increase female representation. However, at least for new drugs, something is slowly changing, especially when only sex differences are considered [6,35,36,37,38], especially in the North American late-phase clinical trials [39,40,41]. However, gender aspects such as environmental (diet, drugs such as valproic acid, air pollution, chromium, arsenic, and microbiota) and psychosocial factors (public speaking task and chronic restraint stress) are even less studied, and their interactions are still ignored, although some of them may alter nuclear and mitochondrial gene expression [42,43,44,45,46].

Therefore, we listed the main sex and gender issues that should be recalled by health professionals and researchers in adopting sex and gender approaches. 

## 2. Factors That Can Affect the Pharmacological Response 

**Figure 1:**
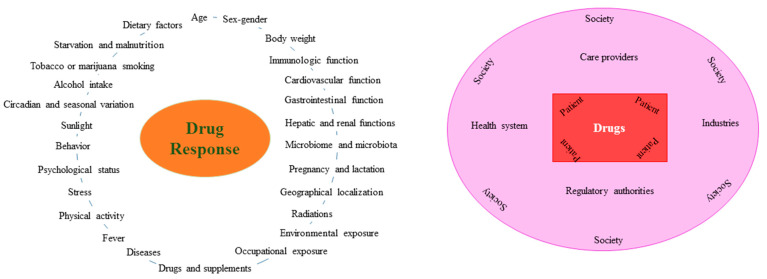
Factors that influence the pharmacological response.

Beyond the characteristics of the drug and the individual (Table 2), which are extensively reviewed [38,47,48], the pharmacological response depends on other numerous factors and their interactions [49], which are summarized in Figure 1. Sex and gender differences involve drug consumption [6,50,51,52,53] and adherence to therapy, which usually tends to be larger in men and women, respectively, with some exceptions [54,55]. 

### 2.1. Influence of Gut Microbiome and Microbiota on the Pharmacological Response 

Globally, the gut microbiota is highly personalized by life experiences, influenced by numerous individual and environmental factors (diet, drugs, etc.), and presents numerous sex and age differences [73,74]. The importance of the gut microbiota in drug response is extensively reported (172) as a modifier of the pharmacokinetics, efficacy, and safety profiles of medications [38,47,75]. In particular, it metabolizes many endogenous and exogenous compounds through its CYP and/or through cooperation with the host CYP enzymes [76]. It is not known as to whether bacterial CYP activity depends on the sex–gender of the host, but, certainly, the mammalian CYP enzymes present numerous examples of sexual dimorphism ([6,75] and cited literature). Beyond CYP enzymes, bacteria also have β-glucuronidase, whose inhibition reduces the activity of nonsteroidal anti-inflammatory drugs ([75] and cited literature). In women, the gut microbiota depends on both endogenous and exogenous (oral contraceptives, OC; hormonal replacement therapy, HRT) sexual hormones. The microbiota of postmenopausal women is more similar to that of men in comparison to that of premenopausal women [73], whereas OC alter both microbial species abundance and functional pathways [73]. The microbiota itself affects estrogen levels; thus, it may be an important regulator of circulating estrogens and estrogenic molecules [73]. The endocrine activities of the intestinal bacteria may be a source of sex–gender differences participating in sex–gender healthcare paradigms [77,78]. 

Further, bidirectional pathways exist between medicines and gut bacteria, and it may mitigate drug side effects, improve or reduce drug efficacy, and control antibiotic resistance; notably, the female gut microbiota presents a higher level of antibiotic resistance genes than the male one [73,75]. 

The gut microbiome is changed by antibiotics and non-antibiotic drug prescriptions such as proton pump inhibitors, metformin, nonsteroidal anti-inflammatory drugs, opioids, statins, and antipsychotics, which are used by millions of people [79]. The possibility of drug-induced changes in the microbiota should be included in sex–gender-based medicine and should be accurately investigated in individuals of different ages. 

Globally, the gut microbiota may generate sex–gender differences, as it is also a modifier of the efficacy and safety profiles of fully participating sex–gender healthcare paradigms [77,78]. From a holistic perspective, it is necessary to take the genetic makeups of the host and microbiome into account when evaluating the role of the microbiota in xenobiotic metabolism, because the microbiota may change the “canonical” pharmacokinetics, changing the clinical effectiveness and safety of the medications. 

### 2.2. Adverse Drug Reactions: “First Do No Harm” (168)

Adverse drug reactions (ADR) have a great impact on health. In Europe, ADR cause five percent of hospital admissions and 197,000 deaths per year [80]. In the USA, ADR are the fourth to sixth highest cause of death [81]. Nevertheless, the paradigm “one dose fits all” is still applied, and women and men receive the same drug dosage for a myriad of diseases, forgetting that sexual biological differences, including hormones, change all pharmacokinetic parameters (Table 2) [38]. Accordingly, it was shown that sex differences in pharmacokinetics predict ADR in women but not in men [82]. The low enrollment of women in clinical trials does not enable assessment of the safety profile of the drug before it goes on the market. Consequentially, the safety profile in women is mainly based on pharmacovigilance, and it may generate several limitations including underreporting, variations in the quality of information, and missing data [83]. In addition, a reporting bias [83], which is not fully calculated [84,85], is present. It has been shown that ADR prevail in women. In particular, the majority of drugs excluded from the market for ADR occurred mainly in women ([6] and cited literature), more women than men are admitted to hospitals for ADR, and more female inpatients develop ADR [86]. An Italian study shows that ADR induced by cardiovascular drugs are more frequent in women than in men [87]. A narrative review concludes that to be of the female sex is a risk factor for cutaneous ADR, major bleeding, etc. [88]. Conversely, some investigations show that fatal and more serious ADR occur more frequently in men than in women ([6,87] and cited literature). Interestingly, an analysis of the Swedish national pharmacovigilance database (2008–2011) shows that women and men have higher rates of nonserious and severe reactions, respectively [89]. Data from VigiBase show that fatal ADR occur more in men, in the elderly over 65 years, and in Americans [90], indicating the importance of sex–gender, age, and geographical localization. Interestingly, paradoxical ADR (which are opposite reactions to the drug’s pharmacological effects) seem to be more frequent in men than in women [91].

Sex and gender differences partly depend on differences in pharmacokinetics and pharmacodynamics; however, the role of the above-mentioned factors in the drug’s safety profiles is not known. However, patient adherence to a therapy or drug prescription depends on the sex and gender of patients and sex and gender of physicians, geographical localization, and microbiota [38,88,90]. 

In conclusion, safety profile also seems to be linked with gender factors.

### 2.3. Ethnicity and Geographical Localization

Ethnicity categorizes people of shared ancestry and physical traits [92] and connotes cultural, linguistic, behavioral, and religious factors [93]. It influences the pharmacological response, and the following examples clarify this point. The use of sodium–glucose cotransporter 2 inhibitors depends on ethnicity, sex–gender, and income, being lower for African Americans and women and higher in higher-income countries [94]. Hypertensive African Americans are more responsive to aldosterone inhibitors, especially women [95,96,97,98,99]. Finally, we recall that cytochrome P450 enzymes (CYP) expression and activities are influenced by sex–gender (Table 2) and ethnicity [100]. The previous examples indicate that drugs should be tested in a specific population and specific sex–gender. 

Geographic location is not considered as stratifying factor, but it can directly affect drug potency [101,102]. A very recent paper reports that administration of propofol and cisatracurium besylate in Han Chinese and Austrian men and women presented some pharmacodynamic and pharmacokinetic differences. Notably, the sex and gender differences were present only in Chinese cohorts [103]. 

Relevantly, it was shown that geographical localization also affects outcomes. For example, the survival one year after a cardiovascular event was greater in men than in women if they live in Southern Europe, while there were no sex–gender differences in the cohorts living in Northern Europe [104]. 

### 2.4. Stress Effects

Stress is part of life, but long and severe stress can negatively affect health, with the hypothalamic–pituitary–adrenal (HPA) axis being crucially involved. In stress situations, females release more corticotropin-releasing factor, arginine vasopressin, adrenocorticotropic hormone, and cortisol, which could be linked to higher sensitivity of corticotropin receptors, whereas negative feedback is higher in males than in females, and the female adrenal cortex can release more stress hormones [105]. 

Chronic stress induces numerous neuroimmune alterations, which in turn produces modifications in neurotransmission and synaptic plasticity within stress-related neural circuitry [106]. Women are more vulnerable to inflammation [107], but the association between stress-related psychiatric illnesses and low-grade inflammation is more frequent in men than in women [108,109]. C-reactive protein, an inflammatory marker, is linked to psychiatric disturbances only in men [109]. In line with the previous study, a Dutch investigation shows that several immune biomarkers associated with depression such as C-reactive protein, trefoil factor 3, cystatin-C, fetuin-A, β2-microglobulin, CD5L, FASLG receptor, and tumor necrosis factor receptor 2 are male-specific [110]. Importantly in HIV subjects, the stress and loneliness induced by the COVID-19 pandemic are higher in women than in men [111].

The psychological stress of different origins such as work, poor-quality or insufficient relationships, poverty, and unemployment. [112,113] may affect pharmacological response. Stress may modify pharmacodynamic targets such as blood glucocorticoid receptor gene in adolescents [114], kappa–opioid receptor in adulthood, and benzodiazepine receptor binding [115]. In addition, stressors may modify the pharmacokinetic elevating hepatic drug metabolic activities [116,117,118,119], probably through the enzyme induction promoted by corticosteroids [120,121,122,123]. Further, stress may also modify gastrointestinal functions, lipid distribution, blood flow, the albumin binding capacity, and renal elimination [124,125]. Interestingly, some of these changes occur in a sex–gender-dependent way such as the modification of adiposity induced by chronic stress that prevails in men [126]. 

The above findings indicate that stress is an important regulator of pharmacokinetics and pharmacodynamics participating in the origin of the interindividual variability of drug responses. This is of particular interest for women because stress, including posttraumatic stress disorders, prevails in women [127,128]. Paradoxically, the majority of investigations on stress were performed in male animals, creating problems in data translation because preclinical data could not be valid for both sexes [129]. Moreover, in depression, where there is chronic stress, clinical trials tend to recruit more women than men. 

It is also important to recall that men’s and women’s life experiences differ in work and life stress [130,131], and this can partially explain differences in levels of disease burnout between men and women [130]. 

### 2.5. Social Events, Socioeconomic Position, Unemployment, and Low Education Levels

The stress response also depends on income, as it is lower in high-income countries than in low/medium-income countries [124,132]. Further, work stress may alter some biomarkers, which often are used to verify the therapeutic response [133]. Given the numerous sex–gender differences in the stress response [127], it is plausible that stress-induced changes can be sex–gender-specific [10]. Accordingly, in women, the stress response is influenced by the menstrual cycle and maternity where oxytocin seems to play a crucial role [134]. 

The effect of stress on pharmacological response is often ignored for the complexity of research and economic costs but it needs to be studied because participates fully in the generation of interindividual variability in a sex-specific way; the awareness of this plays a fundamental role in personalized care. 

### 2.6. A Peculiar Case of Stress Effect: The Caregiver Response to Vaccines

In recent years, the number of caregivers for a loved one with physical and mental deterioration is dramatically elevated and the majority of them are women [135,136]. The burden of stress is higher if they are a spouse, a sole caregiver, and have a lower income [137]. Globally, health problems (especially depression or anxiety and cardiovascular diseases) prevail in women [138,139,140,141,142]. In low-income countries, a recent study evidenced that male caregivers report anxiety when compared to situations where no one is ill in the household [143].

Importantly, 40% of informal caregivers assist in drug administration, but many of them did not receive any training or instructions [144]. 

Both biological and gender factors impact vaccine acceptance, responses, and outcomes [145]. Men and women diverge in vaccine-induced immune responses, adverse events, and protection. For example, following vaccination, women typically have higher antibody responses and more adverse effects compared to men [146]. Only a small amount of sex–gender-stratified data are available for vaccinated caregivers, but the response to the pneumococcal pneumonia vaccine is reduced in caregivers [147]. At four weeks after the influenza vaccination, only 38% of caregivers had a clinically significant antibody response versus 66% of non-caregivers. Finally, after varicella–zoster virus vaccination, poorer cell-mediated vaccine responses were present in caregivers [145]. The differences were more evident in the elderly [145]. Relevantly, acute stress after vaccination may increase vaccine side effects [148]. Given numerous sex–gender differences in vaccine response, the immune system, and the higher percentage of female caregivers, sex and gender differences are more than plausible. 

### 2.7. Stigma 

Stigma is a shameful state of disapproval that occurs in subjective feelings of being rejected and excluded from society [149]. At least in part, it depends on the norms and values that govern everyday life [150]. Stigma involves the entire continuum of care at least in people with mental health diseases such as drug abuse [151]. It can affect subjects in numerous manners, creating discrimination, numerous disadvantages, and problems. Some sex differences were reported in weight stigma [152] and HIV stigma, which was more frequent in women than in men; however, in other countries, men internalized stigma more often than women [153]. In addition, sex differences were reported in drug abusers, but quantitative investigations were not univocal, whereas qualitative investigations evidence that stigma prevails in women [154].

Importantly, stigma increases the stress response, which plays a role in the drug response [155] (see above).

### 2.8. Sex-Gender Differences at the Origin of Life 

Both the mother and the father contribute to the neonates’ genetic makeup; the quality of the fetal environment participates in the trajectory of fetus development [156,157,158]. Numerous maternal risk factors are identified, but paternal factors receive less attention [159]. For example, both paternal and maternal depression may increase the risk for preterm birth [160]. In addition, prenatal maternal psychological distress exerts sex–gender-specific effects on fetuses influencing future psychopathologies, such as possible deregulation of the HPA axis or aberrant brain development [161]. 

Experimentally, the antenatal glucocorticoids modify gene expression in the prefrontal cortex in a sex-specific manner, as extracellular ligand-gated ion channel activity and synaptic signaling are upregulated in females and downregulated in males [162]. These alterations may be transmitted to the successive generation [162]. 

Beyond hormones, drugs and other abused substances may modify the developmental programming, causing alterations in developmental trajectory [163,164]. For example, longitudinal studies reveal that prenatal exposure to psychotherapeutics may lead to future learning disabilities and mental health disorders, even in babies born without birth defects [164]. Globally, exposed male progeny seem to be more susceptible than female progeny [164]. 

Global environmental and social factors may affect the exposed individual and his/her progeny altering the epigenome in a sex–gender manner. This indicates the need for knowing the prenatal and neonatal history of babies, including ART and drug treatments, because they may modify the patient’s phenotype, contributing to the interindividual variability in drug responses [165], and could be useful for stratification in clinical trials. The changes induced by hormones and other molecules can be transmitted to the next generations, indicating they should be used for stratification in clinical trials that will be planned in the future. 

### 2.9. The Professional–Patient Relationship Influences the Therapeutic Response

Social skills strongly influence professional–patient relationships, and this could influence diagnostic and therapeutic decisions [166]. Notably, sex–gender influences the relationship between professionals and patients [38,167,168,169,170]. When male doctors treat patients with myocardial infarction, female patients survive less often than male patients. On the other hand, female physicians appear to achieve the same therapeutic goals, regardless of the patient’s sex [171]. A small but statistically significant decrease in 30-day mortality was observed in surgically operate females by female physicians [6] and in elderly hospitalized patients when treated by female internists [172]. Many other examples can be found in the recent review of Champagne-Langabeer and Hedges [166]. The above data suggest that physician/patient dyads of the opposite sex could change the outcomes, and the benefits of the same sex–gender dyad seem most evident in female patients. 

The influence of the physician sex–gender on drug prescription is not univocal [173,174,175,176]. Female physicians seem to have a more conservative approach in prescribing a drug to older adults [177]. The doctor’s prescribing patterns are also affected; physicians attribute more psychological factors to women than to men, and thus they prescribe more antidepressant and anxiolytic drugs to women than to men [53]. The nonunivocity in prescription could also depend on the specific cultures, religions, etc., of physicians [178]. Prevention, diseases, and mortality may be different through different cultural–religious settings [179,180]. For example, religion can influence the use of contraception in very distinct ways; for Catholics, the use of medical or physical contraceptive methods is forbidden, while there is no religious opposition to any contraceptive method among Protestants or in Confucianism and Taoism. 

In addition, some of previous differences may derive from a rational process that affects our capacity to judge in a rigorous manner or in our non-verbal communications, leading to the so-called implicit bias [181]. Sex–gender implicit bias is worldwide [182] and can influence the behavior of health professionals when they interact with stigmatized people [183]. Male and female healthcare providers may present some divergences in implicit racial bias, being higher in males than in females [184]. 

## 3. Intersectionality 

The term was coined by Kimberlé Crenshaw in 1989, illustrating the need for African American women to consider the intersection of two dimensions of inequality: race and gender [183]. Intersectionality shows that the multifactorial interactions of social factors that induce discrimination when it is not apparent when we exclusively look at one. Intersectionality is now expanding into sex–gender-based health research with the aim to evaluate the interconnections between nature and social categorizations that may create overlapping and interdependent systems of discrimination when applied to a subject or group of persons [47,184]. 

Indeed, the traditional model of medicine, focused on the diagnosis and cure of diseases, does not fit int sex- and gender-based medicine with its specific gender issues such geographical localization, stigma, caregiver role, and professional–patient relationship, whose effects on health and diseases are often neglected. Importantly, intersectionality provides value to social factors intersecting with sex–gender and helps in identifying multidimensional, structural discriminations [185,186,187,188]. Notably, the intersections are related to the contest supporting the idea focusing on a peculiar aspect of diversity that is linked to individual experience to structures and structural discrimination. Therefore, researchers and professional should be trained to recognize the intersecting factors and their effects on the health of individuals.

## 4. Future Perspectives

It is evident that, until now, low economic status, low education levels, discrimination, stressors, microbiota, stigma, religion, access to healthcare, caregiver role, etc., markedly affect health, diseases, treatments, and outcomes. It is well known that some of these adversities interact with gender (intersectionality) [189,190,191]. The concern for sex and gender medicine is becoming popular; this produces some positive actions such as an elevation of awareness among health professionals, institutions, and regulatory agencies; allocation of funds; etc. The high value for health of social determinants is well known [192,193]; however, in sex- and gender-based medicine, poverty, low education levels, underrepresentation of violence, etc., continue to be neglected [194]. However, in some studies, the importance of socioeconomic status both in men and in women was evaluated [195,196,197]. For example, the mortality rate and socioeconomic associations are generally lower in women than men [196]. 

### 4.1. Preparation of Questionnaires 

For this lack of knowledge to be overcome, specific questionnaires could be prepared to be administered in order to collect more relevant information about other health determinants as we suggested some years ago [198]. The questionnaires should be validated by scientific societies. 

### 4.2. Enrollment 

To overcome the lack of scientific knowledge, which generates inequalities, we must enroll women and other gender identities in clinical studies. Investigations should apply a sex- and gender-based analysis as a mandate from the Canadian Institutes of Health Research [199] and should follow the suggestions for the collection and storage of samples, as suggested in Franconi et al. [198].

### 4.3. Research Team 

Men and women should constitute the research teams and care providers because every person is sexed and gendered, regardless of whether they are a researcher or a care provider. Both teams must also have the cultural competence to manage big data without forgetting the numerous ethic issues regarding human health [200]. The clinical research team should be multidisciplinary (physicians, nurses, psychologists, sociologists, pharmacists, biologists, anthropologists, epistemologists, statisticians, computer scientists, patient representatives) to redefine sex- and gender-based medicine, prioritize research and policy topics, and participate in the design of clinical studies. This will facilitate multistakeholder collaboration through the construction of orchestrated common language, which, in turn, will help to reduce the burden of noncommunicable diseases due to a better understanding of environment–biology interactions. 

### 4.4. Research and Health Professional Training 

Researchers and health professionals should be focused on the person and not solely on the disease, considering psychological health and social events and how they can contribute to the prevention, medicine, and treatments [6,201]; biological aspects are largely dependent on interactions with environments [6,48,112,198,202]. Actually, only limited data that investigated this specific point are available. Interestingly, people with dementia in elderly care homes are treated with a great number of psychotropic medications, despite the low effectiveness and safety profiles of these treatments. When individuals with dementia received a person-centered model of dementia, there is a decrease in drug use, including inappropriate medications [203]. Patients with chronic heart failure treated with a person-centered integrated palliative advanced homecare have a higher health-related quality of life and a decrease in hospitalizations than the control group [204]. It is evident that the sanitary policies should provide new rules in order to go beyond the disease, seeing the person’s needs. The universities should be taught a medicine that puts the person in the center of the care.

In addition, researchers and health professionals should acquire the awareness of implicit biases, which could help to elevate the care through mitigation of personal biases [205] and how to apply intersectionality. Professionals such as physicians and nurses should be prepared to know the patient’s psychosocial and cultural contexts [6,32,38,206]. According to Ziegelstein [207], we need a new-omic, namely, “personomics”. In addition, professionals should be trained on the interconnections between nature and social categorizations that may create overlapping and interdependent systems of discrimination.

### 4.5. Building an Alliance 

It is necessary to build an alliance among regulatory and ethic authorities, the healthcare system and health professionals, researchers, the pharmaceutical and diagnostic industries, decision makers, and patients in order to elevate sex–gender-based research and application of sex–gender-based medicine according to the patients’ needs. The alliance could prepare registries with gender approaches after the market introduction of drugs and medical devices that should provide more information on the efficacy and safety of drugs and medical devices [208]. 

## 5. Conclusions

Overcoming the sex–gender bias requires the enrollment of women and other minorities in clinical studies, as well as the use of research designs that include gender aspects because many health inequalities depend on sex and gender [6,22,23,24]. The application of real sex–gender studies is necessary to provide a more appropriate therapy to single subjects and to avoid structural inequalities in healthcare. Here, we underlie that the enrollment of women is a necessary step, but it is not enough because clinical researchers have to adopt sex gender approaches, being aware that gender issues such as stigmatization, caregiving, and implicit biases greatly affect therapeutic response. The application of real sex–gender-based medicine urgently requires data performed with rigorous sex–gender approaches 

## Figures and Tables

**Table 1:** Some sex and gender definitions.

Organization	Sex	Gender
**WHO** [2]	The different biological and physiological males and females characteristics.	Refers to the socially constructed characteristics of women and men, such as norms, roles, and relationships of and between groups of women and men. It varies from society to society and can change, including how they should interact with others of the same or opposite sex within households, communities, and workplaces.
**European Institute of Gender Equality** [1]	Biological and physiological characteristics that define humans as female or male.	Social attributes and opportunities are associated with being female and male and with the relationships between women and men and girls and boys, as well as with the relations between women and those between men.
**National Institutes of Health** [3]	Biological differences between females and males, including chromosomes, sex organs, and endogenous hormonal profiles.	Socially constructed and enacted roles and behaviors, which occur in a historical and cultural context and vary across societies and over time. All individuals act in many ways that fulfill the gender expectations of their society. With continuous interaction between sex and gender, health is determined by both biology and the expression of gender.
**Canadian Institutes of Health Research** [4]	A set of biological attributes in humans and animals. It is primarily associated with physical and physiological features including chromosomes, gene expression, hormone levels and function, and reproductive/sexual anatomy. Sex is usually related to female or male, but there is variation in the biological attributes that comprise sex and how those attributes are expressed.	Refers to the socially constructed roles, behaviors, expressions, and identities of girls, women, boys, men, and gender-diverse people. It influences how people perceive themselves and each other, how they act and interact, and the distribution of power and resources in society. Gender is usually conceptualized as a binary (girl/woman and boy/man), yet there is considerable diversity on individuals and groups understand, experience, and express it.
**Australian Government** [5]	Refers to the chromosomal, gonadal, and anatomical characteristics associated with biological sex.	It is a part of a person’s personal and social identity. It refers to the way that a person feels, presents, and is recognized within the community. A person’s gender may be reflected in outward social markers, including their name, outward appearance, mannerisms, and dress.

Modified from [18].

**Table 2:** Sex differences that can affect PK parameters in adult men and women.

Parameters	Sex Differences
Body weight	higher in M
Gastric secretion (pH)	higher in M (hormone-dependent)
Gastric emptying rate	higher in M (hormone-dependent)
Gastro-intestinal mobility	higher in M (hormone-dependent)
Fat	higher in F (differences are age-dependent)
Muscular mass	higher in M (differences are age-dependent)
Keratinocyte size	higher in M
Skin pore size	higher in M
Total water (intracellular and extracellular)	higher in M
Albumin protein binding	=
Red blood cells	higher in M (it could vary the distribution and metabolism of drugs)
Plasma volume	higher in F
Cardiac output	higher in M
Heart rate	higher in F
Regional blood flow	higher in M
Glomerular filtration rate	higher in M
Creatinine	higher in M
CYP1A2 activity	higher in M
CYP2A6 gene, protein, and activity	higher in F users of OC
CYP2A7 gene	higher in F
CYP2A16 gene	higher in F
CYP2C16 activity	higher in M
CYP2E1 activity	higher in M
Liver CYP3A4 gene, protein, and activity	higher in F
Liver CYP3A5 gene	higher in M
Liver CYP3A7gene	higher in F
CYP2B6 gene, protein, and activity	higher in F
CYP2C9 activity	=
CYP2C19 activity	=
CYP2D6 activity	higher in M
Liver CYP7A1 gene	higher in F
Liver GSTA1/A2 gene	higher in F
UDP-glucuronosyl-transferase 2 expression and activity (human liver)	higher in M
SULT1A1	higher in F than men with high androgen levels
SULT1E1 liver	higher in F =
N-acetyltransferase activity	higher in F
Catechol-O-methyl-transferase activity	higher in M
Liver OATP2, OATP7, expression	=
Liver P-glycoprotein expression and activity	higher in M
Liver breast cancer-resistant protein	higher in M
Liver SLC3A1 gene (encodes neutral and basic amino acid transport protein rBAT)	higher in F
Liver SLC13A1 gene (encodes sodium/sulfate cotransporter)	higher in M
Liver SLC10A1 gene (encodes sodium/bile acid uptake system)	higher in F
Liver ACSL4 gene (encodes Acyl-CoA synthetase long chain family member)	higher in F
MRP	higher in F (differences are age-dependent)

Data from [42,56,57,58,59,60,61,62,63,64,65,66,67,68,69,70,71,72] and cited literature. F = female; M = male; UGT = UDP-glucuronosyl-transferase; SULT = sulfotransferase; SLC = solute carrier family; MRP = multidrug resistance protein; Oatp = organic anion-transporting polypeptide.
